# Microbial underdogs: exploring the significance of low-abundance commensals in host-microbe interactions

**DOI:** 10.1038/s12276-023-01120-y

**Published:** 2023-12-01

**Authors:** Geongoo Han, Shipra Vaishnava

**Affiliations:** Molecular Microbiology and Immunology, Brown University, Providence, RI USA

**Keywords:** Immunology, Mucosal immunology

## Abstract

Our understanding of host-microbe interactions has broadened through numerous studies over the past decades. However, most investigations primarily focus on the dominant members within ecosystems while neglecting low-abundance microorganisms. Moreover, laboratory animals usually do not have microorganisms beyond bacteria. The phenotypes observed in laboratory animals, including the immune system, have displayed notable discrepancies when compared to real-world observations due to the diverse microbial community in natural environments. Interestingly, recent studies have unveiled the beneficial roles played by low-abundance microorganisms. Despite their rarity, these keystone taxa play a pivotal role in shaping the microbial composition and fulfilling specific functions in the host. Consequently, understanding low-abundance microorganisms has become imperative to unravel true commensalism. In this review, we provide a comprehensive overview of important findings on how low-abundance commensal microorganisms, including low-abundance bacteria, fungi, archaea, and protozoa, interact with the host and contribute to host phenotypes, with emphasis on the immune system. Indeed, low-abundance microorganisms play vital roles in the development of the host’s immune system, influence disease status, and play a key role in shaping microbial communities in specific niches. Understanding the roles of low-abundance microbes is important and will lead to a better understanding of the true host-microbe relationships.

## The Mighty Microbes: Low-Abundance Organisms’ Role in Immunity

A new study reveals low-abundance commensals play key roles in host physiology. These microorganisms, including bacteria, fungi, archaea, and protozoa, establish mutualistic relationships with the host and impact various phenotypes such as immune activation, disease occurrence, social behavior, and protection against pathogenic infections. Interactions between these microorganisms and other commensal bacteria in their niche also occur. Further research is needed to explore the potential of low-abundance microbes as disease markers or therapeutic targets.

This summary was initially drafted using artificial intelligence, then revised and fact-checked by the author.

## Introduction

Our understanding of host-microbe interactions has grown based on the work of many researchers in past decades. These studies have revealed the various roles of commensal bacteria in host phenotypes, including obesity and diabetes^1–5^, brain development and behavior^6–8^, protection against pathogenic infection, diseases^9–11^, aging, and response to cancer therapy^12^. However, the majority of investigations primarily focus on the dominant members within ecosystems, typically emphasizing highly abundant bacteria and viruses (including bacteriophages) while neglecting low-abundance microorganisms. This tendency persists, particularly in studies employing high-throughput sequencing approaches^13^. Furthermore, it is important to note that laboratory animals maintained under clean conditions usually lack microorganisms beyond bacteria, such as fungi and helminths. Indeed, the microbiome-associated phenotypes observed in laboratory animals, including the immune system, are markedly different from those observed in the real world because the commensal microbiome present in the natural environment exhibits a considerably more intricate and diverse community structure. As an example, laboratory mice and humans show distinct immune profiles; however, by cohousing laboratory mice with pet-store mice or rewilding laboratory mice, it is possible to recapitulate human immune traits^14–16^. Intriguingly, recent studies have unveiled the beneficial roles of certain helminths, also known as parasitic worms, in host physiology through helminth-bacterial interactions, although we do not cover helminths in this review because they are non-commensal and they are not microorganisms^17–20^. In brief, helminth infection (e.g., *Trichuris muris*) induces immune responses that play a crucial role in maintaining the integrity of the epithelial barrier. These responses involve the activation of type 2 cytokines and IL-22, which synergistically increase goblet cell mucus production and antimicrobial peptide expression. Moreover, helminth infection brings about alterations in bacterial composition that favor a beneficial microbial milieu. Specifically, helminth inhibits the proliferation of inflammatory *Bacteroides* species while promoting the growth of protective bacteria such as *Clostridiales*; this reshaping of the bacterial community contributes significantly to the amelioration of inflammatory bowel disease (IBD) severity.

Recently, numerous studies have directed their attention toward low-abundance microbes within the community. These studies emphasize the fact that low abundance does not mean less important using the “keystone species” concept. Despite their low abundances, these keystone taxa play a pivotal role in shaping the microbial composition and performing specific functions within the host, as supported by recent research^21,22^. Therefore, a better understanding of low-abundance microorganisms is essential to elucidate true host-microbe interactions. In this review, we describe how low-abundance commensal microorganisms interact with the host and contribute to the host’s health. Specifically, we highlight recent important findings on the roles of low-abundance bacteria, fungi, archaea, and protozoa in the context of host phenotypes, including the development of the immune system, the association with diseases, and interactions with commensal bacteria.

## Low-abundance bacteria

In many microbiome studies, low-abundance bacteria (usually referring to a taxon that accounts for less than 1% of the relative abundance) have often been neglected in comparison to high-abundance “dominant” bacteria, primarily due to their rarity in the environment. Furthermore, a significant number of researchers opt to filter out the low-abundance taxa from their microbiome studies (e.g., 16S rRNA gene sequencing analysis) during the quality control and filtering steps prior to downstream analysis^13^. Despite their low abundances, the number of taxa and diversity of low-abundance bacteria is much higher than that of high-abundance bacteria in the host^23^. Multiple studies have consistently emphasized the substantial contributions of low-abundance bacteria to host phenotypes, underscoring the necessity to allocate comparable attention to these less prevalent members alongside dominant members when investigating host-microbiome interactions. In this review, we present up-to-date information on the role of low-abundance microbes in regulating host physiology and disease pathology while highlighting the knowledge gap in this area (Table 1).

**Table 1:** Summary of the roles of low-abundance bacteria in the host.

Microbes	Habitat	Findings	Ref
*Actinobacteria*	Intestine (Human)	Increase in obese individuals than in lean subjects	^40^
^*U*^*Borkfalki ceftriaxensis*	Intestine (Human)	Restoration of the microbiome after antibiotic intervention	^47^
*Eggerthella lenta*	Intestine (Human, Mouse)	↑ Th17 responses in mice Enrichment in IBD patients and contributes to colitis in mice	^27^
		↓Th17 responses in mice; negative correlation with CD patients	^28^
		Association with rheumatoid arthritis in humans	^36^
		Increase in MS patients	^37^
*Erysipelotrichaceae*	Intestine (Mouse)	↑ MHC class II expression on the intestinal epithelium in mice	^23^
*Escherichia*	Intestine (Human)	Contribution to bacterial pilus assembly in the human intestine	^46^
*Helicobacter*	Intestine (Mouse)	↑ MHC class II expression on the intestinal epithelium in mice	^29^
*Mucispirillum schaedleri*	Intestine (Mouse)	Indicator of DSS-induced colitis in mice	^34^
		Increase in an IBD mouse model (A20 knockout mouse)	^35^
		Increase in a social stress mouse model	^38^
		Protection against *Salmonella* infection in mice	^39^
*Ruminococcus*	Intestine (Human)	Marker for enterotype 3 in humans	^46^
Low-abundance taxa	Intestine (Human)	Association with preclinical AD status in humans	^44^
	Oral cavity (Human)	Keystone species in pregnancy gingivitis patients	^41^
	Airway (Human)	Association with CF disease	^45^
	Intestine (Termite)	The driver of compositional alteration after the diet changes	^22^

### Roles of low-abundance bacteria in the host immune system

The pivotal role of commensal gut bacteria in immune development is widely recognized. For example, *Bacteroides fragilis* has been recognized to induce systemic Th1 cells through the utilization of bacterial polysaccharides. Segmented filamentous bacteria (SFB) or *Candidatus* Savagella, previously named *Candidatus* Arthromitus, induce the differentiation of intestinal Th17 cells via the presence of flagellins^24–26^. Notably, even low-abundance bacteria make significant contributions to host immune development.

*Eggerthella lenta*, previously known as *Eubacterium lentum*, is a human commensal in the intestine and is present at a very low level in healthy subjects^27^. Interestingly, this prevalent gut commensal plays opposite roles in the context of immune responses. Alexander et al. reported that *E. lenta* promotes Th17 activities by reducing the inhibition of Rorγt in mice^27^. They also showed that the cardiac glycoside reductase 2 (Cgr2) enzyme encoded by this bacterium is sufficient for IL-17A induction and that dietary arginine blocks *E. lenta*-induced intestinal inflammation. In contrast, another study demonstrated that this particular bacterium possesses the ability to convert lithocholic acid (LCA) into 3-oxolithocholic acid (3-oxoLCA) and isolithocholic acid (isoLCA), which, in turn, suppress Th17 cell differentiation^28^. Moreover, the anti-inflammatory metabolites 3-oxoLCA and isoLCA were inversely associated with Crohn’s disease (CD).

The genus *Helicobacter* is one of the major histocompatibility complex (MHC) class II-associated bacteria in the intestine. It was reported that mice naturally colonized with *Helicobacter* species such as *H. mastomyrinus* and *H. typhlonius* show higher epithelial MHC class II expression than *Helicobacter*-free mice, and the relative abundance of *Helicobacter* species is positively correlated with epithelial MHC class II expression in the mouse intestine. In addition, cohousing of *Helicobacter*-free mice with *Helicobacter*-harboring mice increased MHC class II expression^29^. In our previous study, we demonstrated the potential of low-abundance bacteria in the context of MHC class II expression using a dilution strategy. In this study, the cecal microbiome of mice was diluted to eliminate low-abundance bacteria, and the undiluted and diluted microbiome was engrafted into the intestines of germ-free mice. Loss of low-abundance bacteria resulted in low expression of multiple genes involved in the MHC class II antigen presentation pathway and lower MHC class II-expressing cell counts in the small intestine. Notably, the relative abundance of the family *Erysipelotrichaceae* was positively correlated with MHC class II expression^23^. The findings from multiple independent studies provide consistent evidence of the immunogenic properties exhibited by *Erysipelotrichaceae*^30–32^. Palm et al. classified and selected intestinal bacteria based on immunoglobulin A (IgA) coating. They revealed that high IgA coating is related to colitogenic bacteria and that unclassified *Erysipelotrichaceae* is one of the high IgA-coating bacteria^31^. Dinh et al. showed a positive correlation between the relative abundance of *Erysipelotrichaceae* and tumor necrosis factor alpha (TNF-α)^32^. Taken together, these studies highlight the significant role of low-abundance bacteria in modulating host immunity. Further studies are needed to establish the causal link between these low-abundance microbes and specific immune responses.

### Association between low-abundance bacteria and diseases

Low-abundance bacteria play a crucial role in host disease. An important point is that they play different roles in diseases; sometimes they have a preventive role, but in other situations, they are associated with the disease. *Mucispirillum schaedleri*, which belongs to the phylum *Deferribacteres* and is a low-abundance commensal bacteria in humans and rodents, plays both types of roles^33^. IBD is representative of gut microbiome-associated disease and has been the subject of extensive research concerning host-microbe interactions. Numerous investigations have consistently demonstrated an association between IBD and *M. schaedleri*. This bacterium was identified as an indicator of dextran sodium sulfate (DSS)-induced colitis in mice^34^. A20 functions as a potent inhibitor of both the NF-kB and apoptotic signaling pathways, making it a pivotal gene associated with susceptibility to various inflammatory diseases, including IBD. The abundance of *M. schaedleri* was significantly higher in A20 knockout mice than in wild-type mice^35^. There is another noteworthy IBD-associated bacterium, *E. lenta*, a human commensal with known Th17-modulating properties. This bacterium is enriched in IBD patients and contributes to colitis in a *Rorc*-dependent manner in mice^27^. Furthermore, previous studies have indicated an observed correlation between *E. lenta* and both rheumatoid arthritis (RA)^36^ and multiple sclerosis (MS)^37^. Moreover, mice with social stress have an increased abundance of *M. schaedleri* in the intestine^38^. In contrast, *M. schaedleri* protects against enteric pathogen infection. In the mouse intestine, *M. schaedleri* acted as an antagonist of *Salmonella enterica* serovar Typhimurium virulence. *M. schaedleri* protected the host against *Salmonella* colitis by competing for anaerobic respiration substrates and inhibiting the expression of virulence factor, a type 3 secretion system encoded on *Salmonella* pathogenicity island-1 (T3SS-1)^39^.

The relationship between commensal microbes and metabolic diseases has been reported. One of the most well-known relationships is between obesity and gut microbes, and low-abundance bacteria are associated with obesity. White et al. observed that the phylum *Actinobacteria*, which are known as low-abundance bacteria in the gut, is associated with obesity. In this study, among obese subjects, *Actinobacteria* constituted ~5% of the gut microbiota; meanwhile, these bacteria had a significantly lower prevalence in lean individuals^40^.

The gut microbiome is closely related to various gastrointestinal tract diseases and is considered both a disease determinant and a potential therapeutic target. In the oral cavity, specific groups of low-abundance bacteria, such as *Fretibacterium* sp. OT 361 in subgingival plaque (SGP), *Prevotella_intermedia* in saliva, and *Porphyromonas endodontalis* in SGP and saliva, were identified as keystone species in pregnant patients with gingivitis^41^.

Alzheimer’s disease (AD), the most common cause of dementia, is a neurodegenerative disorder that results in the deterioration of cognitive function^42^. Emerging evidence suggests that the composition of the gut microbiome, alongside the presence of low-abundance bacteria, plays a notable impact on the progression of AD^43^. Ferreiro et al. recently reported that intestinal commensal bacteria can be an indicator of AD in humans^44^. They showed that multiple intestinal low-abundance bacteria, such as *Dorea formicigenerans*, *Oscillibacter* sp. 57_20, *Coprococcus catus*, and *Ruminococcus lactaris*, are associated with a preclinical AD status, whereas *Methanosphaera stadtmanae* is associated with a healthy status.

Furthermore, low-abundance bacteria are key determinants of a healthy airway microbiome early in human life. Pust and Tümmler reanalyzed public shotgun metagenomic data of healthy children and children with cystic fibrosis (CF)^45^. They observed that rare taxa are the most important factors in deciding whether a child is healthy or suffering from life-limiting CF disease. These rare members are essential to improving the underdeveloped CF background network. Collectively, these studies detail the robust relationships between low-abundance bacteria and various diseases. However, most findings have primarily focused on establishing associations rather than elucidating causality or underlying mechanisms. Therefore, it is imperative to conduct consistent and comprehensive investigations to further understand these relationships and to explore their potential therapeutic implications.

### Impact of low-abundance bacteria on the bacterial community

Low-abundance bacteria play a key role mediators of microbial composition. Low-abundance bacteria are usually not considered core members of the intestine; however, Benjamino et al. revealed that they act as drivers of compositional alteration after diet changes. In this study, they provided six different lignocellulose food sources to termites and performed 16S rRNA gene sequencing and artificial neural network analysis to predict the relative abundances of taxa at random points in the termite hindgut. The study reported that low-abundance taxa, which frequently exist outside of the core community, maintain community-driving correlations in the hindgut microbiome^22^. The enterotype study of the human gut microbiome is another example underscoring the role of low-abundance bacteria in maintaining community structure. In this study, the genus *Ruminococcus* was identified as a representative marker for enterotype 3. Notably, the relative abundance of this genus was lower than that of *Bacteroides*, a marker for enterotype 1, and *Prevotella*, a marker for enterotype 2, albeit the relative abundance was slightly higher than 1%^46^. Hildebrand et al. demonstrated the possibility of low-abundance bacteria as keystone species in restoring the gut microbial community after antibiotic intervention^47^. The authors identified a novel bacterial species, initially named ^U^*Borkfalki ceftriaxensis* and subsequently revised as *Candidatus* Borkfalkia ceftriaxoniphila^48^, from the human intestine. This bacterium bloomed to 92% relative abundance after administration of cephalosporin (ceftriaxone) in the intestine and restored the microbial community into a healthy but shifted community state in the long term. Importantly, most species co-occurred with ^U^*B. ceftriaxensis* were probiotic species frequently used to treat antibiotic-associated diarrhea (AAD).

Low-abundance bacteria also play important microbial functions in the community. For example, the genus *Escherichia* includes well-studied bacteria and their abundance is low in the healthy gut; however, the function of this genus should not be ignored. Low-abundance *Escherichia* contributes over 90% of the amount of two abundant proteins related to bacterial pilus assembly, FimA and PapC, in the human intestine^46^. Pili help bacteria stay longer in the intestine and are key components for transferring plasmids between bacteria. These findings underscore the fundamental significance of minor members within the bacterial population, highlighting their crucial role not only in promoting community stability but also in contributing to essential functions.

## Fungi

Fungi are commensal members of the host and ubiquitously exist in diverse body sites such as the intestine, lungs, and skin, and fungal composition is very different based on their niche^49^. However, they are usually considered to be minor members and are not well studied compared to bacteria due to their low abundance (~0.1% of total microbes in the gut)^46,49^. The significance of commensal fungi in host-microbiota interactions has often been underestimated, primarily due to the limited size of fungal databases and the comparatively low abundance of fungal cells within the host when compared to bacteria. However, it is important to note that fungal cells are substantially larger, ~100-fold, than bacterial cells, and they occupy a significant amount of physical space. Moreover, as eukaryotes, fungi contribute distinctive metabolic attributes to both the host and the microbiome^49^. It is worth noting that even minor members of the microbiome can exert profound effects on the host, including via the immune system (Table 2).

**Table 2:** Summary of the roles of fungi in the host.

Microbes	Habitat	Findings	Ref
*Candida albicans*	Intestine (Human) Intestine (Mouse; with host adaptation)	Activation of CD8^+^ T cells Protection against influenza A virus infection	^56^
		↑ Th17 responses in humans and mice	^57,58^
		↑ Granulopoiesis in the mice’s bone marrow Protection against MRSA infection in mice	^59^
		Induction of macrophages-mediated antifungal IgG production	^60^
		Increase in IBD patients	^55,68,69^
		Association with asthma development	^78^
*Candida parapsilosis*	Intestine (Mouse)	Promotion of diet-induced obesity in mice	^70^
*Candida tropicalis*	Intestine (Mouse)	Migration of RALDH^+^ DCs to the peripheral lymph nodes	^63^
*Malassezia restricta*	Intestine (Human)	Induction of IL-17A and IFN-γ-producing CD4^+^ T cells in the colon Increase in IBD patients and colitis mouse model	^61^
*Malassezia*	Intestine (Human, Mouse)	Increase in PDA tumors in humans and mice Increase in CRC patients	^85,86^
*Mucor racemosus* *Mucor fuscus*	Intestine (Human)	Decrease in obese subjects	^71^
*Pichia kudriavzevii*	Intestine (Human)	Association with asthma development	^76,77^
Mucosa-associated fungi	Intestine (Human, Mouse)	Protection against DSS-induced colitis and *Citrobacter* infection Promotion of social behavior	^62^
Wild mycobiome	Wild environment	Expansion of circulating SSC^hi^ granulocytes and neutrophils Increase of multipotent progenitors	^16,59^
Intestinal fungi	Intestine (Human, Mouse)	Association with liver diseases	^80,81^

### Roles of fungi in the host immune system

Commensal fungi have been recognized for their close association with the host’s immune system, and among them, *Candida albicans*, a commensal fungus in humans, has emerged as a widely employed model organism for investigating host-fungal interactions. Although *C. albicans* typically colonizes as a commensal in healthy individuals, under conditions of microbial dysbiosis or in individuals with compromised immune systems, this fungus transitions to a pathogenic state, leading to significant mucosal and systemic infections^50,51^. The formation of *C. albicans* hyphae is a pivotal characteristic linked to the transition from a commensal to a pathogenic state, as hyphae exhibit increased invasiveness and virulence. In the commensal state, most *C. albicans* exists in the yeast form, with only a limited presence of hyphae^52^. The host epithelium plays a barrier function and separates *C. albicans* from the host, collaborating with the immune system and commensal bacteria to maintain homeostasis^51^. The mucus layer and commensal bacteria actively contribute, through both direct and indirect mechanisms, to the physical separation of *C. albicans* from the epithelium. Consequently, impairment of the epithelial barrier or bacterial dysbiosis can facilitate *C. albicans* infection. Furthermore, epithelial cells actively modulate the commensal state of *C. albicans* by initiating immune responses. These immune responses encompass the activation and recruitment of innate immune cells, including neutrophils, monocytes, and macrophages, as well as the production of secretory IgA (sIgA) and β-defensins, which are triggered by the release of IL-22 from Th17 cells or innate lymphoid cells. Notably, these immune reactions exhibit a preferential induction of the hyphal form of *C. albicans*^53–55^.

Numerous studies have consistently highlighted the capacity of commensal *C. albicans* to induce certain immune responses in both human and murine hosts. Mono-colonization of bacteria-depleted mice with either *C. albicans* or *Saccharomyces cerevisiae* in the intestine positively calibrated the activation of protective CD8^+^ T cells and protected the host against influenza A virus infection. Moreover, treatment with mannans, a fungal cell wall component, recapitulated the protection of these two commensal fungal species^56^. Furthermore, the intestinal colonization of *C. albicans* has been shown to elicit Th17 responses in both humans and mice^57,58^. *C. albicans* was chosen as a main inducer of human Th17 responses, and cross-reactive Th17 cells against *C. albicans* contributed to *Aspergillus fumigatus*-driven non-intestinal inflammation^57^. In mice, intestinal colonization by *C. albicans* induced systemic Th17 responses and protected the host against *C. albicans* invasive infection as well as *Staphylococcus aureus* invasive infection^58^. *C. albicans* colonization in the gut also enhances granulopoiesis. Oral inoculation of laboratory mice with *C. albicans* induced persistent expansion of myeloid progenitors in the born marrow dependent on IL-6R signaling. This expansion consequently conferred host protection against systemic methicillin-resistant *Staphylococcus aureus* (MRSA) infection as well as intraperitoneal or intranasal infection with *Streptococcus pneumoniae*^59^.

Human gut commensal mycobiota, primarily *C. albicans*, induce CARD9^+^CX3CR1^+^ macrophage-mediated antifungal immunoglobulin G (IgG) production and protect the host against systemic *Candida* infection^60^. *Malassezia restricta*, a commensal fungus found on the skin, is another example of an immune-stimulating fungus. Intestinal colonization of specific-pathogen-free (SPF) mice with this particular fungal species induced upregulation of IL-17A and IFN-γ-producing CD4^+^ T cells in the colon^61^. A distinct fungal community inhabits the intestinal mucosa of humans and mice, whereby these mucosa-associated fungi (MAF) induce Th17-mediated immune responses^62^. MAF, including *C. albicans*, *S. cerevisiae*, and *Saccharomycopsis fibuligera*, protected the host against DSS-induced colitis and *Citrobacter rodentium* infection via IL-22-dependent mechanisms. Furthermore, MAF promote social behavior by activating IL-17-mediated signaling in neurons. The gut mycobiome also mediates immune cell migration. Zhang et al. reported that commensal fungi are responsible for inducing the migration of CD45^+^CD103^+^RALDH^+^ dendritic cells (DCs) to the peripheral lymph nodes after birth^63^. Intestinal colonization of both SPF and germ-free (GF) mice with *Candida tropicalis* triggered the migration of CD45^+^CD103^+^RALDH^+^ DCs from the lamina propria of the intestine to the inguinal and mesenteric lymph nodes. These DCs utilized retinoic acid signaling to initiate an increase in lymph node cellularity and volume expansion.

As mentioned above, *C. albicans* is the most studied fungal species in the context of commensal-immune interactions. Nevertheless, the use of *C. albicans* as a model commensal fungus in mice presents a certain limitation. Specifically, *C. albicans* is not a natural murine commensal, which forces the modulation of intestinal bacterial communities through antibiotic treatment or the use of GF mice to establish successful *C. albicans* colonization, and this modified, bacteria-free environment is not the natural habitat of commensal fungi. Given the limitations of *C. albicans* as a murine commensal fungus, many researchers have undertaken efforts to investigate the impact of murine commensal fungi on the immune system in mice. One of the strategies is using host-adapted strains of *C. albicans*. Rahman et al. isolated *C. albicans* strain 529 L using a low-estrogen murine model of concurrent oral and vaginal *C. albicans* colonization^64^, and this strain exhibited stable colonization in the mouse intestine^65^. Another strategy is rewilding SPF laboratory mice, wherein they are exposed to natural environments, enabling interactions with diverse microbes, including fungi, that they would not encounter under controlled laboratory conditions. Rewilded mice displayed increases in activated T cells and circulating neutrophils with notable increases in the colonization of intestinal fungi. Notably, colonization of SPF mice with fungal consortium isolated from wild mice—*Aspergillus candidus*, *Aspergillus proliferans*, *Chaetomium globosum* and *Dichotomopilus indicus*—was sufficient to induce expansion of circulating SSC^hi^ granulocytes and neutrophils^16^. In rewilded mice, intestinal fungal colonization resulted in a notable increase in the quantity and frequency of multipotent progenitors (MPPs), particularly within the myeloid-biased MPP3 subset that resides in the bone marrow^59^. Collectively, these findings highlight the significance of the fungal microbiome in the context of immune development. Therefore, it is imperative to incorporate the study of the mycobiome when investigating interactions between the immune system and the microbiome.

### Association between fungi and diseases

The association between commensal fungi and host diseases has consistently been reported^66,67^. As we mentioned before, IBD is one of the most actively studied diseases in the context of host-bacteria interactions. Commensal fungi also show significant involvement in the pathogenesis of this disease. In IBD patients, reduced fungal diversity and increased relative abundance of *Candida* species in the gut were observed^68^. Furthermore, the analysis revealed an elevated *Basidiomycota*/*Ascomycota* ratio, a reduced proportion of *S. cerevisiae*, and an augmented proportion of *C. albicans* in comparison to the fungal composition of healthy individuals^69^. The human intestinal mycobiota, which includes *C. albicans*, stimulates the production of sIgA, with the induced sIgA exhibiting a preference for binding to hyphae, a fungal morphotype commonly associated with virulence^55^. In CD patients, the antifungal sIgA titer was lower with increased granular hyphal morphologies when compared to healthy individuals. *M. restricta* is another IBD-associated fungal species. *M. restricta* was more abundant in CD patients than in healthy controls. In particular, patients carrying the CD-linked polymorphism in CARD9, CARD9^S12N^, were strongly associated with *M. restricta*, and intestinal colonization of this fungus exacerbated DSS-induced colitis in mice^61^.

Obesity is also related to gut commensal fungi, and the supplementation of antifungals such as amphotericin B or fluconazole can effectively inhibit high-fat-diet-induced obesity in mice^70^. The commensal gut fungus *Candida parapsilosis* is a strong candidate for obesity-associated fungal species. This fungus increased free fatty acids in the gut through the production of fungal lipases, and it promoted diet-induced obesity in mice^70^. On the other hand, *Mucor racemosus* and *M. fuscus* were more abundant in nonobese subjects than in their obese counterparts in the human gut^71^.

Asthma is a chronic airway disease, and evidence of the association between the composition of bacteria in the intestine and lung and childhood asthma has been reported^72–74^. In addition, recent studies have indicated that asthma is linked not only to commensal bacteria but also to commensal fungi^75–78^. Arrieta et al. analyzed the fecal microbiome of Ecuadorian babies using 16S and 18S rRNA gene sequencing^76^. They observed that microbial dysbiosis at 3 months of age is associated with later atopic wheeze development that includes fungal dysbiosis. Despite no differences in the alpha- and beta-diversity of the fungal community between healthy control and atopic wheeze groups, atopic wheeze subjects showed a distinct fungal composition characterized by an overgrowth of *Pichia kudriavzevii* and a reduction in *Saccharomycetales* compared to the healthy control infants. The same research group replicated these study findings in Canadian babies^77^. In comparison to the healthy control group, subjects with asthma symptoms showed an overgrowth of *P. kudriavzevii* in the intestine at 3 months of age. Furthermore, neonatal intestinal overgrowth of *P. kudriavzevii* exacerbated the manifestations of type-2 and type-17 inflammation during allergic airway disease in subsequent stages in mice. *Candida* species are also asthma-associated fungal candidates. In the house dust mite (HDM)-induced mouse asthma model, oral administration of *C. albicans* led to increased airway inflammation, including increased total white cell and eosinophil counts in the airway and total immunoglobulin E (IgE) concentrations in the serum^78^. In addition, *Candida*-colonized mice showed no fungal burden but an increase in the abundance of group 2 innate lymphoid cells (ILC2s) compared to the control in the lungs. In humans, patients experiencing severe asthma exacerbation exhibited an elevated *Candida* burden in the intestine.

The gut–liver axis refers to the reciprocal interaction between the gastrointestinal tract, its resident microbiome, and the liver, resulting from the integration of signals generated by dietary, genetic, and environmental factors^79^. Intestinal fungi, a part of the gut microbiome, are also associated with liver diseases^80^. Yang et al. observed that chronic alcohol consumption increases the intestinal fungal burden and the level of circulating β-glucan, a fungal cell wall component, in mice^81^. Treatment with amphotericin B, an antifungal agent, reduced intestinal fungal burden and the level of circulating β-glucan and ameliorated the symptoms of alcoholic liver disease (ALD). The authors observed that β-glucan elicits hepatic inflammation through the C-type lectin-like receptor, CLEC7A, present on Kupffer cells, and the subsequent increase in IL-1β, a proinflammatory cytokine, actively contributes to hepatocyte impairment and facilitates the progression of ethanol-induced liver disease. In ALD patients, a less diverse fungal community and overgrowth of *Candida* were observed compared with healthy individuals. Intriguingly, *Saccharomyces boulardii*, a probiotic yeast that is not gut commensal, exhibits prominent hepatoprotective effects in D-galactosamine-induced liver injury and nonalcoholic steatohepatitis^82–84^.

The association between commensal fungi and cancer has also been revealed. Pancreatic ductal adenocarcinoma (PDA) tumors in both humans and a mouse model display an approximately 3000-fold elevation in fungal burden when compared to normal pancreatic tissue^85^. The mycobiome in PDA tumors showed a composition distinct from that of gut or normal pancreatic tissue. *Malassezia* species demonstrated remarkable enrichment within the tumor microenvironment and promoted oncogenesis by driving the activation of the complement cascade via mannose-binding lectin (MBL) activation. MBL specifically recognizes glycans present on the fungal cell wall, thus contributing to the accelerated progression of malignancy. Gao et al. reported fungal dysbiosis in fecal samples from polyp and colorectal cancer (CRC) patients^86^. This dysbiosis was characterized by decreased diversity in polyp patients, an increased *Ascomycota*/*Basidiomycota* ratio, and an increased proportion of *Trichosporon* and *Malassezia* compared to healthy controls. On the other hand, fungal-derived cell wall components can be utilized in cancer therapy. For example, BTH1677 is a fungal-derived, water-soluble, 1,3–1,6 β-glucan, and this particular agent elicits synchronized anticancer immune responses when administered in conjunction with antitumor antibody therapies^87^. Taken together, these studies provide compelling evidence regarding the association between commensal fungi and various diseases. The identification of these associations may provide valuable insights into the potential utility of commensal fungi as disease markers or therapeutic targets.

## Archaea

Archaea are components of the commensal microbiome and are ubiquitously present in our bodies, including in the intestine, vagina and oral cavity^88^. Although archaea are often overlooked in microbiome research due to their distinct biological characteristics^89^, their roles in the host immune system and diseases have been consistently reported (Table 3).

**Table 3:** Summary of the roles of archaea and protozoa in the host.

Category	Microbes	Habitat	Findings	Ref
Archaea	*Methanobacteriales*	Intestine (Human)	Association with obesity	^97^
	*Methanobrevibacter oralis*	Oral cavity (Human)	Association with periodontitis	^94^
	*Methanobrevibacter smithii*	Intestine (Human)	Increase in MS patients Maturation of DCs	^91,92^
	*Methanosphaera stadtmanae*	Intestine (Human)	Maturation of DCs; association with childhood asthma Association with IBD	^92,95,96^
	*Natrinema* sp. J7-2	Intestine (Human)	Increase in CRC patients	^98^
Protozoa	*Blastocystis*	Intestine (Human)	↑ Bacterial diversity with alteration of bacterial composition	^106,107^
	*Entamoeba*	Intestine (Human)	Association with bacterial diversity	^108^
	*Tritrichomonas*	Intestine (Mouse)	↑ Tuft cells and subsequent induction of ILC2 responses	^103–105^
	*Tritrichomonas musculis*	Intestine (Mouse)	Protection against *Salmonella* infection in mice Exacerbation of the development of colitis and CRC	^101,102^

### Roles of archaea in the host immune system

*Methanobrevibacter smithii* is the most dominant methanogenic archaea in the human gut, and this methanogen interacts with immune systems^90^. In MS patients, the relative abundance of *M. smithii* was increased in patients with elevated breath methane compared with healthy subjects^91^. Furthermore, *M. smithii* exhibited positive correlations with several genes, namely, MAPK14, MAPK1, LTBR, STAT5B, CASP1, and HLA-DRB1, which are known to play roles in the maturation of DCs, interferon signaling, and triggering receptor expressed on myeloid cells (TREM) signaling pathways. Bang et al. observed that *M. smithii* and another commensal methanogenic archaea, *Methanosphaera stadtmanae*, interact with monocyte-derived DCs (moDCs)^92^. This interaction led to an increase in the cell-surface expression of CD197 and CD86, which play crucial roles in providing costimulatory signals essential for moDC maturation. The role of archaea in regulating host immunity is a relatively unexplored area. More studies are needed to identify archaea species that consistently colonize mammalian hosts and affect their physiologies.

### Association between archaea and diseases

The associations between archaea and diseases have been reported by several researchers, including the aforementioned association between *M. smithii* and MS^88,93^. As an example, *Methanobrevibacter oralis* is highly present in the oral cavity of periodontitis patients^94^. Another archaea-associated disease is asthma. In a cross-sectional analysis utilizing fecal samples obtained from a subset of children enrolled in the KOALA birth cohort study in The Netherlands, conducted at ages 6 to 10 years, a significant inverse association was observed between childhood asthma and intestinal archaea, particularly *M. stadtmanae*^95^. In addition, it has been suggested that intestinal colonization of archaea promotes gastrointestinal and metabolic diseases such as IBD, obesity, and CRC. Lecours et al. reported an association between IBD and *M. stadtmanae*. In this study, IBD patients showed higher *M. stadtmanae* abundance in the intestine than controls^96^. Moreover, stimulation of mononuclear cells with *M. stadtmanae* increased TNF production. The association between archaea and obesity was reported by Zhang et al.^97^. The authors compared the gut microbiome of normal weight, morbidly obese, and post-gastric-bypass surgery individuals and observed a significantly higher amount of the order *Methanobacteriales*, a H_2_-oxidizing methanogen, in obese individuals than in other groups. Shotgun metagenomic analysis revealed that CRC patients show a distinct intestinal archaea profile compared with healthy individuals^98^. Fecal samples from CRC patients had significant enrichment of halophilic *Natrinema* sp. J7-2 and depletion of methanogenic archaea, including *Methanosphaera*, *Methanococcoides*, *Methanocorpusculum*, *Methanocaldococcus*, and *Methanobacterium*. The role of archaea in the context of mutualism remains largely unexplored, yet these findings emphasize the imperative need to investigate the associations of archaea with diseases.

## Protozoa

In the past, protozoa were primarily considered parasites (e.g., *Cryptosporidium* spp., *Giardia intestinalis*, *Entamoeba histolytica*, *Trichomonas vaginalis*, and *T. tenax*)^99,100^; however, recent research has indicated the existence of commensal protozoa and their role in the host (Table 3).

### Roles of protozoa in the host immune system

*Tritrichomonas musculis*, a murine commensal protozoan, exhibits protective effects against pathogenic infection in mice^101,102^. Chudnovskiy et al. reported that *T. musculis* triggers the activation of the host epithelial inflammasome, leading to subsequent induction of IL-18 production^101^. Inflammasome-driven IL-18 promoted Th1 and Th17 immunity through interferon regulatory factor 8 (IRF8)- and IRF4-dependent DCs, contributing to protection against *Salmonella* Typhimurium infection. In contrast to the findings of these probiotic effects, *T. musculis* exacerbated the development of colitis and CRC. Chiaranunt et al. demonstrated that *T. musculis*-mediated modulation of host immune responses requires the involvement of the NLRP1B and NLRP3 inflammasomes, which collectively confer host protection against *Salmonella* infections^102^. Commensal protozoa increase tuft cells in the intestine, consequently leading to an augmentation of ILC2 populations^103–105^. Tuft cells, also known as taste-chemosensory cells, are characterized by their infrequent occurrence within the intestinal epithelium. These specialized cells exhibit a distinctive ability to respond to protozoan and helminthic stimuli. In response to commensal *Tritrichomonas* species, intestinal tuft cells exhibited an increased abundance, along with the upregulation of IL-25 expression. Notably, this process was contingent upon the cation channel TRPM5-dependent mechanisms and subsequently induces ILC2 responses^103^. Tuft cells express the succinate receptor GPR91 (also known as SUCNR1), which enables sense and response to succinate. Remarkably, immune sensing of *Tritrichomonas* colonization by tuft cells required the succinate receptor, and *Tririchomonas*-derived succinate was sufficient in triggering ILC2 responses^104,105^. Based on these findings, it becomes evident that commensal protozoa play a distinct role in modulating the immune system. These results highlight the necessity for ongoing and consistent investigations into commensal protozoa-immune interactions.

### Impact of protozoa on the bacterial community

Commensal protozoa interact with commensal bacteria and alter bacterial composition in their habitat. In particular, *Blastocystis* spp. colonization led to distinct bacterial composition in the gut^99,106,107^. *Blastocystis*-colonized individuals show increased bacterial diversity with higher abundances of the following taxa: *Clostridia*, *Faecalibacterium*, *Prevotella* 9, *Ruminococcaceae* UCG-002, *Muribaculaceae*, *Rikenellaceae*, *Acidaminococcaceae*, *Phascolarctobacterium*, and *Ruminococcaceae* UCG-005, and lower abundances of the following taxa: *Enterobacteriaceae*, *Enterococcus* species, *Lactobacillales*, and *Bacilli* compared to *Blastocystis*-free subjects. Morton et al. also showed that the presence of the gut commensal protozoa *Entamoeba* is strongly associated with bacterial diversity in rural non-industrialized populations^99,108^. These findings underscore the critical importance of conducting multi-kingdom interaction studies to better understand host-commensal interactions in a comprehensive manner.

## Conclusion

**Fig. 1:**
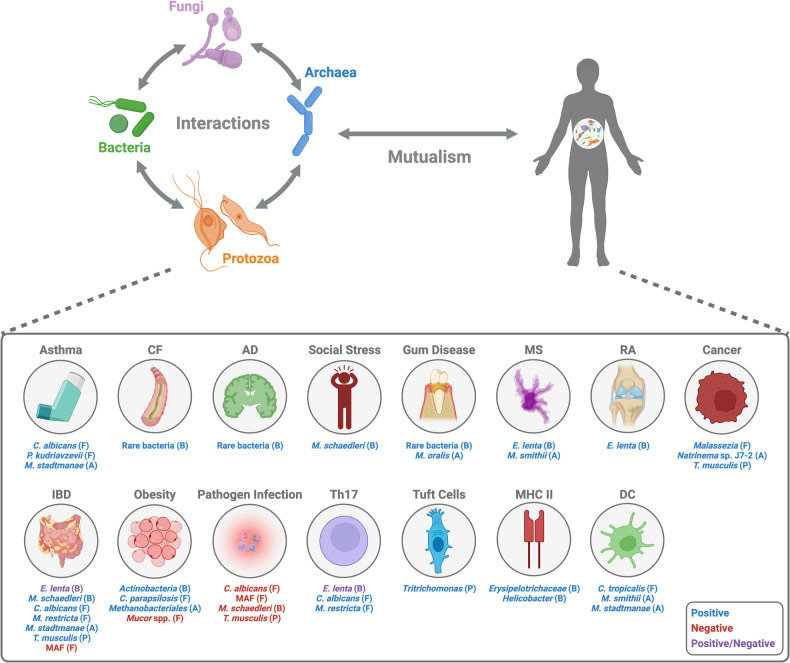
Overview of the significance of low-abundance commensals in host physiology. Low-abundance commensals, including bacteria, fungi, archaea, and protozoa, play pivotal roles in various aspects of host physiology. These microorganisms establish mutualistic relationships with the host, exerting profound effects on the host. Notably, they influence diverse phenotypes, including immune activation (e.g., Th17 cells, tuft cells, dendritic cells, MHC class II expression), the occurrence of several diseases (e.g., inflammatory bowel disease, obesity, asthma, cystic fibrosis, Alzheimer’s disease, multiple sclerosis, rheumatoid arthritis, periodontal disease, cancer), social behavior, and protection against pathogenic infections. Moreover, interactions occur among these microorganisms themselves or with other commensal bacteria in their niche. Created with BioRender.com.

Low-abundance microorganisms, including low-abundance bacteria, fungi, archaea, and protozoa, establish colonization within the animal body and play vital roles in maintaining mutualistic relationships. These microorganisms contribute to the development of the host’s immune system, influence disease status, and play a key role in shaping microbial communities within their niches (Fig. 1). Investigating the roles of low-abundance microbes is imperative; however, researchers must be cautious of potential pitfalls when conducting such studies. First, it is important to acknowledge that the relative abundances of bacteria are inconsistent and variable, both among individuals and even within individuals, particularly in the context of humans and nonlaboratory animals; this fact emphasizes the idea that low-abundance microbes are not always low abundant^109^. Various factors, including diet, genetic background, and the administration of medications such as antibiotics, significantly influence the composition of microbial communities. Therefore, it is crucial to design robust experiments that incorporate a larger sample size with repeated measurements and diverse backgrounds. This approach is essential for elucidating the significance of low-abundance microbes and identifying potential “biomarkers”. Second, while many studies have highlighted the contribution of individual microorganisms, it is important to recognize that microbial communities are inherently intricate and multifaceted rather than being characterized by simplicity. The interspecies interactions within the same microbial kingdom or between different kingdoms yield different outcomes from those observed in single-species colonization in the context of the roles of microorganisms in the hosts^110–112^. Third, it is crucial to take into account the absolute abundance of microbes within each body site. The intestinal tract, for instance, harbors an abundance exceeding one billion microorganisms, whereas other organs harbor a comparatively lower microbial population. Consequently, the utilization of relative abundances alone may yield disparate numerical representations. In this review, we primarily focus on elucidating the significance of commensal low-abundance microbes within the host. However, to gain a comprehensive understanding of the entirety of host-microbe interactions, it is equally important to explore the reciprocal aspect of this relationship—the role of the host, including the immune system, in shaping a mutualistic association with commensal microbes.
